# Interventions targeting the mental health and wellbeing of care-experienced children and young people in higher-income countries: Evidence map and systematic review

**DOI:** 10.1186/s13643-023-02260-y

**Published:** 2023-07-01

**Authors:** Rhiannon Evans, Sarah MacDonald, Rob Trubey, Jane Noyes, Michael Robling, Simone Willis, Maria Boffey, Charlotte Wooders, Soo Vinnicombe, G. J. Melendez-Torres

**Affiliations:** 1 DECIPHer, School of Social Sciences, Cardiff University, SPARK, Maindy Road, Cardiff, CF24 4HQ UK; 2 Centre for Trials Research, Cardiff University, Cardiff, UK; 3 School of Medical and Health Sciences, Bangor University, Bangor, UK; 4 Specialist Unit for Review Evidence, Cardiff University, Cardiff, UK; 5 The Fostering Network in Wales, Cardiff, UK; 6 Peninsula Technology Assessment Group (PenTAG), University of Exeter, Exeter, UK

**Keywords:** Systematic review, Scoping review, Mental health, Wellbeing, Foster care, Residential care, Children, Adolescents

## Abstract

**Background:**

The mental health and wellbeing of care-experienced children and young people (i.e. foster care, kinship care, residential care) is poorer than non-care-experienced populations. The Care-experienced cHildren and young people’s Interventions to improve Mental health and wEll-being outcomes Systematic review (CHIMES) aimed to synthesise the international evidence base for interventions targeting subjective wellbeing, mental health and suicide amongst care-experienced young people aged ≤ 25 years.

**Methods:**

For the first phase of the review, we constructed an evidence map identifying key clusters and gaps in interventions and evaluations. Studies were identified through 16 electronic databases and 22 health and social care websites, in addition to expert recommendations, citation tracking and screening of relevant systematic reviews. We charted interventions and evaluations with a summary narrative, tables and infographics.

**Results:**

In total, 64 interventions with 124 associated study reports were eligible. The majority of study reports were from the USA (*n* = 77). Interventions primarily targeted children and young people’s skills and competencies (*n* = 9 interventions), the parental functioning and practices of carers (*n* = 26), or a combination of the two (*n* = 15). While theoretically under-specified, interventions were largely informed by theories of Attachment, Positive Youth Development, and Social Learning Theory. Current evaluations prioritised outcomes (*n* = 86) and processes (*n* = 50), with a paucity of study reports including theoretical descriptions (*n* = 24) or economic evaluations (*n* = 1). Interventions most frequently targeted outcomes related to mental, behavioural or neurodevelopmental disorders, notably total social, emotional and behavioural problems (*n* = 48 interventions) and externalising problem behaviours (*n* = 26). There were a limited number of interventions targeting subjective wellbeing or suicide-related outcomes.

**Conclusions:**

Future intervention development might focus on structural-level intervention theories and components, and target outcomes related to subjective wellbeing and suicide. In accordance with current methodological guidance for intervention development and evaluation, research needs to integrate theoretical, outcome, process and economic evaluation in order to strengthen the evidence base.

**Systematic review registration:**

PROSPERO CRD42020177478.

## Background

Children and young people with experience of living in care represent a diverse population, with significant international variation in nomenclature and classification [1]. They can be defined as individuals who have had statutory involvement, whereby parental rights have been transferred to another adult. In some countries, such as the UK, there are specific mechanisms to support care entry, such as the issuing of Special Guardianship Orders [2]. Care can include a range of placement types, such as formal kinship care, foster care and residential care [3]. There are also variations in the identity of care-leavers, who are largely defined by their ongoing rights to statutory provision. For example in Germany, young people from a range of care placements are entitled to legal assistance until 21 years old while in England they are entitled to certain services up to 25 [3]. Globally, the estimation of children and young people in care has been challenging, with most recent efforts to establish the prevalence of individuals in institutional care reporting a range from 3.18 million to 9.42 million, depending on the methods and data sources employed [4].

While not a clearly defined population, evidence reports that care-experienced individuals generally have poorer mental health and wellbeing, and higher rates of suicide attempts, compared to non-care-experienced groups [5–9]. Individuals with a history of care have excess mortality in adulthood, attributable to non-natural causes of self-harm, accidents, and other mental health and behavioural risk [10]. Mental health problems incur substantial health and social care costs, largely due to the associated risk of placement instability and breakdown [11–13], which is concerning given increased financial pressures on social care systems [14].

There has been significant development in international intervention research to target reported issues. A number of literature and systematic reviews have synthesised the evidence base for social and healthcare approaches [15–25], with recent National Institute for Health and Care Excellence (NICE) reviews and associated guideline recommendations endorsing implementation of interventions centred on mentoring, positive parenting practices and system change to facilitate more efficient implementation [26].

Despite their contributions, there are two key limitations associated with extant syntheses, relating to both scope and methodology. The first limitation is a focus on a limited range of countries [26]; specific diagnosable conditions (e.g. depression) [20, 21]; discrete population subgroups (e.g. foster care) [17, 20, 27]; or single intervention packages (e.g. Treatment Foster Care) [27, 28]. Where reviews are inclusive of diverse outcomes, populations and intervention types, they tend to take an aggregative approach when presenting syntheses. Notably, there is limited differentiation between the evidence for interventions that operate in different parts of the social system.

This differentiation is imperative, as there is suggestion that interventions can be ineffective due to an over-reliance on individual-level approaches that are minimally disruptive [29], and there is a need to understand the evidence for structural interventions to guide the development of system-level approaches moving forward. Equally, with the advance of complex systems thinking perspectives in intervention research, there is increased recognition that an intervention’s functioning is dependent on its interaction with proximal and distal system characteristics [30–34]. As such, interventions operating in different parts of the system may be subject to different contextual influences and implementation challenges. We need to disentangle these complex interactions to inform effective intervention delivery in future.

There are a number of organising frameworks to help locate interventions in different parts of the social system, including the socio-ecological model, with versions originating from child development and public health research [35, 36]. There are broadly five domains of factors that influence outcomes, and which may be targeted for intervention [35]. These are as follows: intrapersonal, which is an individual’s knowledge, attitude and behaviour; interpersonal, which is an individual’s relationships and social network systems, including family and friendship networks; organisational, which is the formal and informal rules, ethos and characteristics of social institutions; community, which is the relationship between organisations and networks; and policy, which includes local, regional and national laws and policies.

The second limitation with existing reviews is that they tend to restrict syntheses to outcome evaluations, with scant attention paid to interventions’ programme theory, the context of evaluation, the process of implementation, acceptability or cost-effectiveness. Even recent comprehensive NICE reviews [26], which do include a range of evidence types, do not provide a clear overview of programme theories or the contextual factors that give rise to reported barriers and facilitators to intervention functioning.

Integration of these different evidence types is important in understanding how interventions operate and generate effects within their delivery context, and their potential transportability to other health and social care systems. This integrated approach to evaluation, which draws on a range of evidence, is recommended by a range of methodological guidance on intervention development, adaptation and evaluation [37–40]. As such, an evidence map and review that systematically charts the range of interventions targeting the mental health of care-experienced children and young people, in addition to the types of evidence currently generated, is important in identifying where there may be limitations in current intervention research and where it needs to be further strengthened [41].

The Care-experienced cHildren and young people’s Interventions to improve Mental health and wEll-being outcomes Sytematic review (CHIMES) was a complex systems informed, multi-method review that aimed to synthesise international evidence on programme theory, process evaluation, outcome evaluation, equity harms, and economic evaluation [42]. For the first phase, reported presently, we constructed a map of interventions and associated evaluations to chart key evidence gaps and clusters. It addressed the following review questions:

- What are the targeted socio-ecological domains, theories and outcomes addressed in mental health and wellbeing interventions for care-experienced children and young people?
- What are the types of evidence generated as part of intervention evaluations?

In charting the available evidence on interventions and types of evidence, the map informed the scope and feasibility of the second phase of the systematic review. For example, the map identified sufficient randomised controlled trials to conduct meta-analysis for relevant outcomes. The second review phase involved method-level syntheses for outcome evaluations, process evaluations, equity harms and economic evaluations. These were then integrated into an overarching review-level synthesis, where data from one synthesis (e.g. process evaluation) supported explanation of another synthesis (e.g. outcome evaluation) [43]. The third and final phase of the review entailed stakeholder consultation to reflect on the synthesis and prioritise interventions for future development and/or adaptation, evaluation and implementation.

## Methodology

We generated an evidence map, drawing on systematic mapping guidance [44]. Evidence maps have some conceptual overlap with scoping reviews, but with clearer emphasis on stakeholder involvement in the early stages of the research process, a systematic search strategy, and the visual presentation of data [41]. As there is no standardised methodology for the reporting of evidence maps, we describe the process with reference to the Preferred Reporting Items for Systematic Reviews and Meta-Analysis Extension for Scoping Reviews (PRISMA-ScR) [45]. The review is registered with the PROSPERO registry of systematic reviews (reference number CRD42020177478).

### Stakeholder consultation

We integrated a comprehensive programme of stakeholder involvement throughout the CHIMES review. Regarding the evidence map, we consulted with three key groups of stakeholders at the outset to refine and confirm the focus and scope of the review. These groups were as follows: (1) CASCADE Voices (young people’s research advisory group with care-experienced individuals up to the age of 25 years); (2) The Fostering Network in Wales Young Person Forum (group of care-experienced young people who provide advice and guidance to the charity on their programmes of work); and (3) All Wales Fostering Team Managers Forum (group of Local Authority and independent foster care providers). The central priorities of these groups were to focus on wellbeing and suicide-related outcomes, and to map interventions according to a socio-ecological schema. This latter priority related to stakeholders’ perception of a lack of structural interventions at the organisational and policy level, and a need to establish if this is a significant evidence gap and how it might be addressed moving forward.

### Eligibility criteria

The inclusion parameters for the review were defined according to the Population, Intervention, Comparator, Outcome and Study Design (PICOS) framework:

#### Types of participants

Intervention participants could be care-experienced children and young people ($\le$ 25 years old), or their proximal relationships, organisations and communities. Care could include in-home care and out-of-home care (foster care; residential care; and formal kinship care), and could be current or previous (e.g. care-leaver). The amount of time in care was not restricted. The following populations were excluded: general population; children and young people classified as being in need but not placed in care (e.g. having a Children in Need (CiN) plan or Child Protection plan); children and young people at the edge of care; care without statutory involvement; adoption; or unaccompanied asylum seekers and refugees.

#### Intervention

We defined interventions as an attempt to disrupt existing practices in any part of the social system (e.g. healthcare, social care, education, youth justice). They could operate across the following socio-ecological domains: intrapersonal; interpersonal; organisational; community; and policy. They could be mono-component or multi-component. There were no a priori criteria for implementation (i.e. delivery setting, delivery mode, delivery agent). Pharmacological interventions were excluded.

#### Comparator

For outcomes evaluations, a comparator had to be specified and could include: treatment as usual; other active treatment; or no specified treatment.

#### Outcomes

Interventions had to target one of three domains of primary outcomes: subjective wellbeing (in addition to life satisfaction and quality of life); mental, behavioural or neurodevelopmental disorders as specified by the International Classification of Disease (ICD)-11; and suicide-related outcomes (self-harm; suicidal ideation; suicide). Measurement could be dichotomous, categorical or continuous. Outcomes had to be obtained for the child or young person, but could be ascertained through clinical assessment, self-report or report by another informant. Excluded primary outcomes included substance misuse and eating disorders, which have some conceptual overlap with the eligible outcomes, but are large literatures that could form the basis of separate reviews. We mapped all secondary outcomes included in eligible study reports (e.g. physical wellbeing).

#### Study design

Different study designs were eligible according to the research question targeted. Study reports could describe an intervention’s programme theory; outcome evaluation (Randomised Contolled Trial (RCT) or non-randomised design); process evaluation that reported on context, implementation and/or acceptability (qualitative and quantitative design); and economic evaluation (cost-minimisation; cost-effectiveness; cost utility; or cost–benefit analysis).

### Information sources and search strategy

We identified study reports from sixteen electronic bibliographic databases: Applied Social Sciences Index and Abstracts (ASSIA); British Education Index; Child Development & Adolescent Studies; CINAHL; Embase; Education Resources Information Center (ERIC); Cochrane Central Register of Controlled Trials; Cochrane Database of Systematic Reviews; Health Management Information Consortium (HMIC); International Bibliography of the Social Sciences; Medline; PsycINFO; Scopus; Social Policy & Practice; Sociological Abstracts; and Web of Science. We identified additional peer-reviewed studies and grey literature through searching websites of 22 relevant social and health care organisations. Searches were conducted May–June 2020 and updated April–May 2022. We contacted 32 subject experts and 17 third sector organisations for recommendations, particularly regarding grey literature and in progress studies. We screened relevant systematic reviews and conducted forward and backward citation tracking with included study reports. The search strategy was developed in Ovid Medline and adapted to the functionality of each platform (Supplement A). Searches were undertaken from 1990 to coincide with the ratification of the United Nations Convention on the Rights of the Child [46]. Study reports were restricted to higher-resource countries. They were not restricted by language.

### Data selection

We uploaded retrieved citations to the Evidence for Policy and Practice Information and Coordinating (EPPI) Centre’s review software EPPI Reviewer version 4.0 for storage and management. Study titles were screened by one reviewer to identify clearly irrelevant retrievals, with irrelevant reports checked by a second reviewer. Title and abstracts were screened independently and in duplicate by two reviewers. Where there was a conflict on exclusion, the study report progressed to the next stage of screening. Full texts were screened independently and in duplicate with conflicts resolved through discussion or recourse to a third reviewer. An inclusion criteria proforma guided selection, which was tested and calibrated with a subset of retrievals. The same inclusion criteria were applied to study reports from databases and grey literature. Study quality or publication process (e.g. peer review) was not part of the inclusion criteria and was assessed as part of quality appraisal.

### Data extraction

We coded eligible study reports for the evidence map according to country; publication date; intervention type; target population; intervention name; intervention characteristics; programme theory; evidence type; study design; and intervention outcome domains. Intervention characteristics were further coded in accordance with the Template for Intervention Description and Replication (TIDieR) Checklist for Intervention Development [47]. To support description of interventions, we extracted programme theory with a tool used in a previous systematic review [48]. Extraction domains were as follows: method or process for developing the theory; name of theory; discipline of theory; socio-ecological domain of theory; and description of theory.

### Evidence map

Scoping review and systematic mapping methods supported the mapping of the evidence base [44, 49]. Following the coding of study reports, we constructed numerical and narrative summaries of intervention and evidence clusters and gaps, with accompanying infographics. For details on intervention characteristics, we produced a narrative summary and table describing the features according to extractable domains of the TIDieR framework. For interventions reporting on programme theory, we narratively summarised these according to the socio-ecological domains in which they operated and produced a summary table. For evidence types, we constructed a narrative summary and table.

## Results

### Study characteristics

**Fig. 1:**
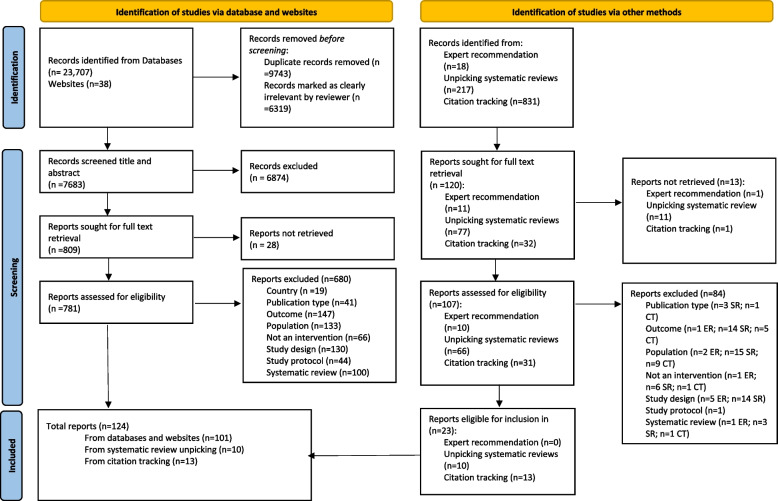
PRISMA flow diagram

A total of 15,068 unique study reports were identified. Of these, 888 were screened at full text, with 64 interventions being included that linked to 124 study reports (Fig. 1) [50–173].

Study reports were published between 1994 and 2022. Interventions were from twelve countries: USA (*n* = 77); UK (*n* = 22); Netherlands (*n* = 6); Belgium (*n* = 3); Australia (*n* = 3); Portugal (*n* = 3); Canada (*n* = 2); Ireland (*n* = 2); Israel (*n* = 2); Germany (*n* = 1); Spain (*n* = 1); Sweden (*n* = 1); and both the USA and UK (*n* = 1).

### Intervention types

**Fig. 2:**
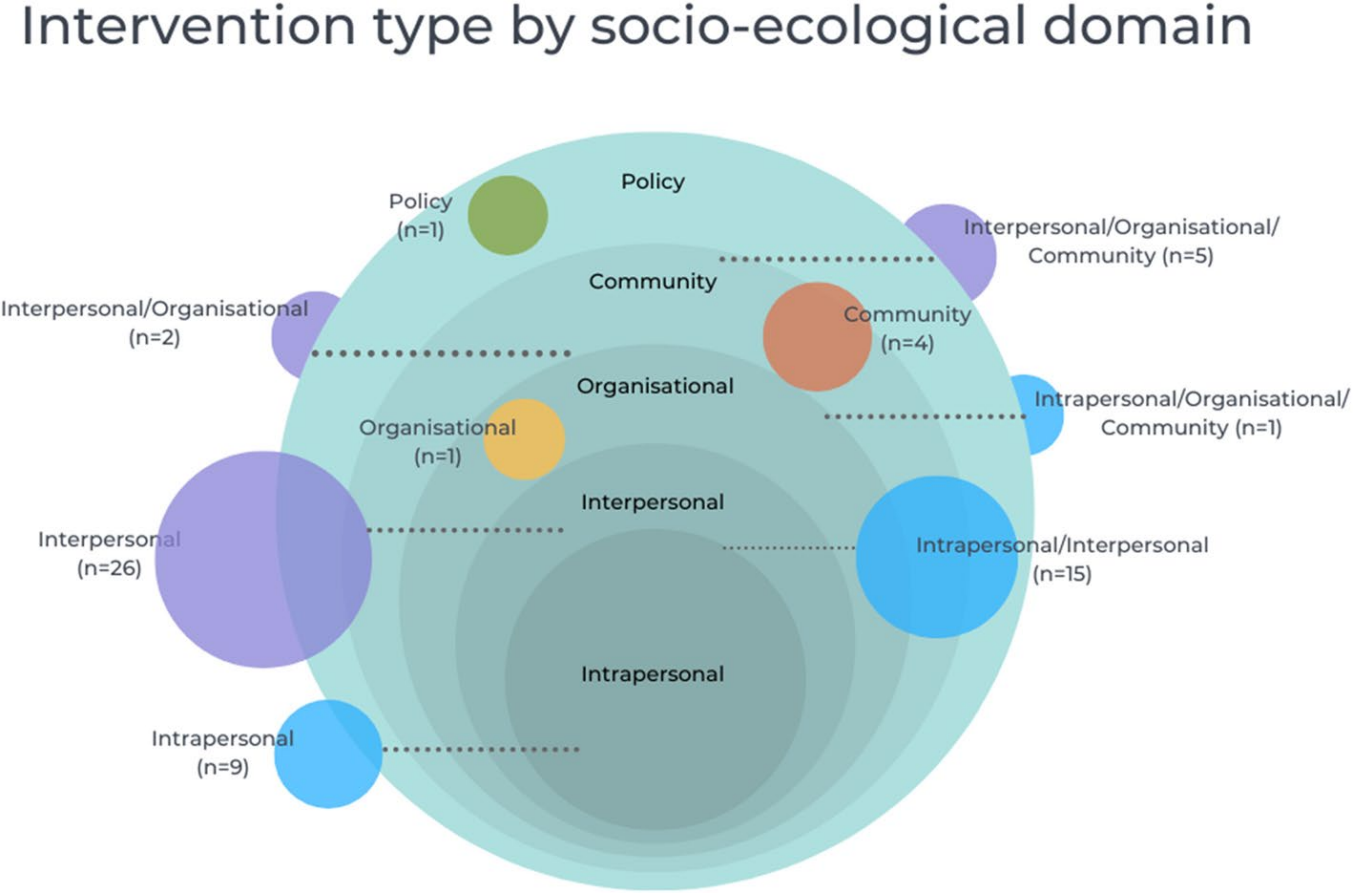
Intervention type by socio-ecological domain

We classified interventions according to the socio-ecological domain or domains targeted (Fig. 2). As indicated, this was due to our assumption that interventions will interact with context differentially if they target different parts of the social system. The classification of interventions by socio-ecological domain was informed by information about the causes being targeted and the reported theoretical basis. Where the theory was not specified, we also drew upon reported information on the target population (e.g. individuals in a relationship with the care-experienced child) and delivery setting (e.g. a social care organisation). While interventions within each group had a shared target set of causes and theories, there was diversity in terms of activities. An overview of intervention characteristics is presented in Table 1.

**Table 1:** Description of intervention characteristics (*N* = 64) [174]

Intervention	Socio-ecological domain	Country	Participant group; Participant age	Intervention duration	Delivery agent and setting	Intervention components
Acceptance and Commitment Therapy (ACT) [155]	Intrapersonal; Interpersonal	Sweden	Children: Residential care Age: 16–18 years	6 weeks	Treatment assistants Residential care placement	Group-based psychoeducational curriculum (2-h sessions) that include experiential exercises, role play and illustrations
Animal Associated Psychotherapy (AAP) [158]	Intrapersonal; Interpersonal	Spain	Children: Residential care Age: Mean 15.17 years	12 weeks	Psychiatrists; Child psychologist Caserio (farm)	Individual and small group sessions (32 sessions) over 2-day visits to a farm. Sessions involve spending time with dogs, horses and other farm animals
Attachment and Behavioural Catch-up (ABC) [143]	Interpersonal	USA	Children: Foster care Age: 0–5 years Adults: Foster carers	10 weeks	Child psychologist; Psychiatric nurse; Social worker Foster care placement	Manualised parenting programme and coaching sessions. Sessions video-taped to tailor content to specific needs of each carer-child dyad. Monthly family support group
Behavioural modification training; psychodynamic treatment; structured community living; adventurous learning [163]	Intrapersonal; Interpersonal	Netherlands	Children: Residential care Age: Mean 14.9 years	Not specified	Not specified for all interventions	Four models of treatment: behavioural management by online care workers; psychodynamic treatment; structured boundaries and relationships; and adventurous learning that models self-supportive, adaptive behaviours
Care placement type [150, 153, 157, 159]	Policy	Netherlands; USA	Children: Foster care; Kinship care; Residential care Age: < 18 years	Not specified	Foster carers; Kinship carers; Residential carers Care placement	Comparison of different types of care placement
Child Adult Relationship Enhancement [135, 167]	Interpersonal	USA	Children: Foster care Age: 3–12 years Adults: Foster carers	Not specified	CARE trainer Not specified	Trauma-informed parenting training (2 × 3 h)
Child and Adolescent Mental Health Services (CAMHS) [80]	Community	UK	Children: Foster care; Residential care	Not specified	Local authority staff; Mental health workers; Psychologists; Psychiatrists Community mental health services	Development of a single referral pathway to improve access and effective engagement with children and young people, through joint professional forums, partnership working and training
Child-Directed Interaction Training (CDIT) [138]	Interpersonal	USA	Children: Kinship care Age: 2–7 years old Adults: Kinship carers	4 weeks	Graduate students Neighbourhood resource centre	Group-based coaching of carers (twice weekly)
Children and Residential Experiences (CARE) [73, 151]	Organisational	USA	Children: Residential care Age: 7–18 years Adults: Residential carers; Social care/health care professionals	3 years	Care Consultants; CARE Implementation Team (CIT) Residential care setting	Consultation, training and technical assistance to residential placements to create a therapeutic environment through policies and practices
Cognitive and Affective Bibliotherapy [123]	Intrapersonal	Israel	Children: Residential care Age: 6–15 years	Not specified	Trained bibliotherapists Residential care placement	Eight small group sessions (45 min) to explore eight texts. Discussion of the texts serves as a departure point for discussing emotions
Cognitively-Based Compassion Training (CBCT) [96]	Intrapersonal	USA	Children: Foster care Age:13–17 years	6 weeks	Not specified	Cognitive training programme (1 h twice per week)
Computer game [74]	Intrapersonal	UK	Children: Residential care Age: 12–17 years	6–18 months	Social worker Online	Online game (6 × 1-h sessions) including SIMS Life Stories (or ‘electronic dolls house’) and emotional regulation skills coaching by a social worker
Connect-KP [117]	Interpersonal	Australia	Children: Kinship care Age: 8–16 years Adults: Kinship carers	9 weeks	Psychologists; Clinical psychologists; Social workers Community settings	Support group sessions (weekly) to develop trauma-informed parenting and explore challenges of kinship care
Dojo: Biofeedback videogame [97]	Intrapersonal	Netherlands	Children: Residential care Age: Mean 13.67	4 weeks	Researcher; Research assistant Online	Online game (30-min sessions twice weekly) with tutorials and emotion-evoking mini-games. Tutorials teach CBT relaxation techniques and positive thinking
Early Intervention Foster Care (EIFC) [56]	Intrapersonal; Interpersonal	USA	Children: Foster care Age: < 6 years Adults: Biological parents; Foster carers	6–9 months	Clinician; Foster carer; Psychiatrist; Psychologist Care placement; School; Day care; Telephone	Before receiving a child, foster carers complete intensive training. After placement, carers are given support thorough daily contact with foster carer consultant, weekly support group meeting and 24-h on call crisis intervention. Children receive services from behavioural specialist and weekly therapeutic playgroup sessions
Enhanced Foster Care Treatment [152]	Interpersonal	USA	Children: Foster care Age: 14–18 years Adults: Foster carers	Not specified	Not specified Foster care placement	Increased financial resource for foster carers and access to educational support
Equine-Facilitated Psychotherapy (EFP) [50]	Intrapersonal; Interpersonal	Israel	Children: Residential care Age: 14–18 years	7 months	Treatment facility staff Treatment facility	Psychotherapy with horses (50 min sessions weekly) to provide a healing experience and develop interpersonal adaptation skills
Evolve Behaviour Support Services (EBSS) [107]	Interpersonal; Organisational; Community	Australia	Children: Out-of-home care; Adults: Biological families; Out-of-home carers; Social care/health care professionals	Not specified	Child Safety Officers; Clinicians Not specified	Holistic and flexible positive behaviour support for children and young people with disabilities, including: child-focused therapy; carer education and training; and environmental strategies. Collaborative working with a range of stakeholders to ensure coordinated, integrated and targeted service delivery
Family Finding [110, 146, 154]	Interpersonal	USA	Children: Foster care Age: 6–17 years Adults: Biological parents; Other family members	40 days	Independent professional; Child’s case worker Not specified	Approach for searching for, discovering and engaging actual and fictive kin to support attachment and permanency needs
Family Minds [120]	Interpersonal	USA	Adults: Foster carers	6 weeks	Study author Online	Group-based curricula (3 classes of 3 h each) to increase carers’ reflective functioning and mentalisation skills
Foster carer and foster children group-based intervention [142]	Interpersonal	USA	Children: Foster care Age: Mean 11.54 years Adults: Foster carers	12 months	Trained foster carers; Graduate students; Undergraduate students	Group-based programme (6 sessions, twice weekly over 3 weeks), with one group for girls and one for foster carers. Follow-up training and support provided (2 h once per week) on individual basis for girls and group basis for carers for subsequent school year
Foster carer training [136]	Interpersonal	UK	Children: Foster care Age: 5–16 years Adults: Foster carers	1 week	Social workers Not specified	Training sessions (6 h per day for 3 days) including didactive material, group material and homework tasks to improve carers communication and attachment
Foster carer training [61]	Interpersonal	UK	Adults: Foster carers	3 days	Clinical psychologist Not specified	Pre-training materials and in-person group training on managing challenging behaviour, with follow-up to discuss progress
Foster parent training [67, 68]	Interpersonal	Belgium	Adults: Foster carers	10 weeks	Trained specialist foster carers Foster care placement	Weekly home visits to deliver a curriculum that includes psychoeducation, practice of emotions and communication skills. Homework tasks of daily 10-min play activity. Group sessions to provide peer support
Fostering Changes [88, 137]	Interpersonal	UK	Adults: Biological parents; Foster carers	12 weeks	Trained facilitators Not specified	Group-based training (3 h weekly) with support group to reinforce learning
Fostering Connections [92, 156]	Interpersonal	Ireland	Adults: Foster carers	6 weeks	Trainer practitioners Community setting	Trauma-informed psychoeducational programme (3.5 h weekly). Content includes experiential exercises, videos, role play, discussion and at home exercises. Carers receive a toolkit and homework book
Fostering Healthy Futures (FHF) [64–66, 69, 70, 101, 144]	Intrapersonal; Interpersonal	USA	Children: Out-of-home care Age: 9–11 years	30 weeks	Facilitators; Masters-level social workers Not specified	Group-based manualised curricula (1.5 h weekly) and one-to-one mentoring to model positive social relationships
Fostering Individualised Assistance programme (FIAP) [125]	Intrapersonal; Organisational; Community	USA	Children: Foster care Age: 7–15 years Adults: Biological parents; Foster carers; Social care/health care professionals	Not specified	FIAP family specialists Foster care placement; Community settings; School	Specialist acts as a family-centred, clinical case manager and home-based counsellor. Provide strength-based assessment, life domain planning, clinical case management, and tailoring of services
Glasgow Infant Family Team (GIFT); London Infant Family Team (LIFT); New Orleans Model [103, 175]	Interpersonal; Organisational; Community	UK	Children: Foster care; Kinship care Age: 0–5 years Adults: Biological parents; Foster carers; Kinship carers; Social care/health care professionals	12 weeks	Psychiatrists; Psychologists; Social workers; Family liaison workers Not specified	Referrals made to multi-disciplinary team who engage in series of interviews, observations and questionnaires to assess family functioning (biological family) and parental mental health to make placement decision. Social work team meets with family (2 h per meeting for eight meetings) over 3 months
Head, Heart, Hands [93]	Interpersonal; Organisational	UK	Adults: Foster carers; Social care / health care professionals	Not specified	Social pedagogues Foster care system	One-day taster session, 2-day orientation course, 8-day core course and follow-on group to support introduction of social pedagogic learning
Head Start [131]	Community	USA	Children: In and out-of-home care Age: 3–4 years Adults: In and out-of-home carers	12 months	Community service providers Community services	Wraparound community services and support, including early learning in vocabulary and early literacy, maths skills, and behavioural and emotional problems
*Health*RHYTHMS [124]	Intrapersonal; Interpersonal	USA	Children: Residential care Age: 12–18	6 weeks	Trained facilitator; Counsellor Not specified	Group sessions (1 h weekly) including self-expression with a drum, before progressing to verbal and written communication. Combined with tactile conditioning, where young people have a crystal to heighten emotional awareness
Herts and Mind: Mentalization-Based Therapy [94]	Interpersonal	UK	Children: Foster care Age: 5–11 years Adults: Foster carers	12 weeks	CAMHS Targeted Team Not specified	Short manualised treatment including a combination of psychoeducation about attachment and mentalising in children with histories of maltreatment; consultations with professionals; and relational work
Incredible Years [60, 82, 84, 87, 98]	Interpersonal	Portugal; UK; USA	Children: Foster care; Kinship care; Residential care Age: 2–12 years Adults: Biological parents; Foster carers; Kinship carers; Residential carers	12–18 weeks	Experienced foster carers; Social workers Care placement; Community settings	Parenting group (2–2.5 h sessions). One-to one home visit programme to reinforce skills learnt during sessions
Individual therapy and rehabilitative strategies [160]	Intrapersonal	USA	Children: Foster care	Not specified. Data availability for 3 years	Counsellors and therapeutic specialists Foster care placement; Therapeutic setting	Individual therapy: Varying types of therapy with mental health provider offering at home sessions 2–5 times per week Therapeutic behavioural services: Treatment services between a child and mental health provider 2–5 times per week
Intensive Permanence Systems (IPS) [86]	Interpersonal; Organisational	USA	Children: Foster care Adults: ‘Supportive connections’	24 months	IPS experienced staff Not specified	Family search and engagement strategies to create a supportive network for youth to help on the path to permanency
*k*Contact [169]	Interpersonal	Australia	Children: Foster care Age: 0–14 years Adults: Biological parents	9 months	Caseworkers Telephone	Four phases of support for biological parents to plan for, reflection upon and review goals for contact with child
Keeping Foster and Kinship Parents Supported and Trained(KEEP) [53, 62, 79, 81, 106]	Interpersonal; Organisational; Community	USA	Children: Foster care; Kinship care Age:4–16 years Adults: Foster carers; Kinship carers; Social care/health care professionals	16 weeks	Trained facilitators Care placement; Community settings	Parenting group (90 min weekly), home practice activities and weekly check in phone calls. Different community implementation models to integrate into child welfare system
Kids in Transition to Schools (KITS) [148]	Intrapersonal; Interpersonal	USA	Children: In and out-of-home care Age: 4–6 years	7 weeks	Not specified Care placement; School	Therapeutic playgroups (2 h, twice weekly) to learn and practice the social and self-regulatory requirements of school
Kundalini Yoga [105]	Intrapersonal	UK	Children: Residential care Age: Mean 14.78 years Adults: Residential staff	20 weeks	Not specified Residential care placement	Yoga classes (44–60-min sessions) teaching posture, breathing and meditation
Life Story [85]	Intrapersonal; Interpersonal	USA	Children: Foster care Age: 7–15 years	7 months	Teachers; Child welfare professionals; Counsellors Foster care placement	Meeting once per week to construct a culturally sensitive narrative of personal experience, where the professional challenges assumptions about substance use
Mentoring intervention for teenage pregnancy [59]	Interpersonal	UK	Children: In and out-of-home care Age: 5–16 years	1 year	Peer mentors Range of settings	Peer mentoring sessions with trained peers. Engaged in a range of activities, and communication via email, face-to-face, telephone and text
Mindfulness [89]	Intrapersonal	USA	Children: Foster care; Kinship care Age: 14–21 years	10 weeks	Psychologist; Research Assistant Health clinic	Mindfulness curriculum (2-h sessions) with guest speakers, crafts, yoga, music and socialising
Multidimensional Treatment Foster Care (MTFC); Multidimensional Treatment Foster Care-Adolescents (MTFC-A); Multidimensional Treatment Foster Care-Pre-schoolers (MTFC-P) [51–53, 58, 76, 78, 90]	Interpersonal; Organisational; Community;	Netherlands; UK; USA	Children: Foster care; Kinship care; Residential care Age: 3–17 years Adults: Foster carers; Social care/health care professionals	Approx. 2 years	Foster carers; Clinicians; Therapists; Programme supervisors; Skills workers; Education workers Care placement; Community settings	Specialist, supported foster carers with expertise in behaviour management. Range of wraparound services, including clinical and educational provision. Different community implementation models to integrate into child welfare system
Nonviolent Resistance (NVR) Training [145]	Interpersonal	Belgium	Children: Foster care Age: Mean 11.6 years Adults: Foster carers	10 weeks	Foster care workers Foster care placement	Training programme (75 min sessions per week) with telephone support between every two sessions, a workbook, handout and DVD,
Opportunities Box [164]	Intrapersonal	Portugal	Children: Foster care Age: 14–17 years	6 weeks	Psychologist Not specified	Sessions (90 min) on career ability, adaptability and decision-making
Outpatient mental health services [147]	Community	USA	Children: Foster care	Not specified	Outpatient mental health service providers Outpatient mental health services	Access and availability to outpatient mental health services, including drug and alcohol clinics, community health centres, crisis centres and private professional treatment
Parent–Child Interaction Therapy (PCIT) [77, 134, 165, 172]	Interpersonal	USA	Children: Foster care Age: 2–7 years Adults: Biological parents; Foster carers	14 weeks	Clinician; PCIT graduate student Outpatient setting; Telephone	Parent management training (2/3 full days and weekly phone consultation) with two stages: Child-Directed interaction (CDI) to promote parent–child bonding; and Parent-Directed Interaction (PDI) to enhance parent management
Parent Management Training (PMT); Parent Management Training Oregon Model (PMTO) [100, 114, 118]	Interpersonal	Netherlands; USA	Children: Foster care; Kinship care Age: 3–16 years Adults: Biological parents; Foster carers; Kinship carers	16 weeks	Trained facilitators Community setting; Care placement	Group programme (90 min 1–2 time weekly) and home visit supervision in behaviour management. Combined with home practice assignments
Pathways Home [54]	Interpersonal	USA	Children: Foster care Age: 5–12 years Adults: Biological parents	32 weeks	Trained consultants Not specified	Parenting curriculum to prevent reunification failure by supporting development of a safe and nurturing environment. Booster sessions to fine-tune skills
Promoting First Relationships [139]	Interpersonal	USA	Children: Foster care Age: 10–24 months Adults: Biological parents	10 weeks	Community mental health agencies Biological parent home	Brief manualised sessions (60–75 min weekly) with video feedback, worksheets and handouts
Psychosocial rehabilitation [166]	Intrapersonal; Interpersonal	USA	Children: Foster care Age: 3–18 years	12–24 months	Children’s psychosocial rehabilitation specialists Foster care placement	Home support offering individualised family-focused and child-centred treatment (4–8 h per week), in addition to specialist support to relevant adults (2 h per week)
Sanctuary Model [162]	Intrapersonal	USA	Children: Residential care Age: 12–20 years	12 weeks	Residential care staff Residential care placement	Psychoeducational curriculum. Technical assistance from residential care staff. Twice daily community meetings
Solution Focused Parenting Group (SFPG) [102]	Interpersonal	Canada	Children: Foster care Adults: Foster carers	6 weeks	Facilitator Not specified	Parenting group (90 min weekly) focusing on identifying parenting solutions, homework to practice skills, and feedback
SuppOrting Looked after children In Decreasing Drugs, and alcohol (SOLID) [72, 108, 109]	Intrapersonal; Interpersonal	UK	Children: Foster care; Kinship care; Residential care Age: 12–20 years	35 days	Motivational interviewing practitioner; Counsellor Care placement	Two behaviour change interventions: Motivational enhancement therapy: Client-centred counselling (6 sessions) with problem feedback component to reflect on impact of drug and alcohol use Social behaviour and network therapy: Behavioural and cognitive strategies to help build social networks that are supportive of positive behaviour change in relation to problem substance use and goal attainment
TAKE CHARGE [113]	Intrapersonal; Interpersonal	USA	Children: Foster care Age: 14–17 years	12 months	Coaches; Peers; Foster care alumni Not specified	Coaching (50 h) in self-determination and goal achievement. Three mentoring sessions with intervention peers and foster care alumni
Teach Your Children Well (TYCW) [133]	Intrapersonal; Interpersonal	Canada	Children: Foster care Age: Not specified	30 weeks	Researchers; Foster carers Foster care placement	Individual tutoring (3-h sessions) including tutoring in reading, reading aloud to foster carer or other adult, and self-paced supervised maths instruction
Therapeutic Mentoring [171]	Interpersonal	USA	Children: Foster care Age:6–15 years	6–9 months	Clinician Not specified	Therapeutic mentoring relationship (4–5 h, weekly) involving pre-planned activities within the mentor–mentee relationship
Trauma-Focused Cognitive Behavioural Therapy (TF-CBT) and evidence-based engagement strategies [83, 112]	Intrapersonal; Interpersonal	USA	Children: Foster care Age:6–15 years Adults: Foster carers; Kinship carers	Not specified	Clinician; Counsellors; Social workers Not specified for TF-CBT; Telephone and foster care placement for engagement strategies	Sessions (12–20 sessions) with child, carer and child-carer, focusing on parenting, psychoeducation and trauma. Supplementary engagement component, with contact between clinician and family via telephone, in person or both to address participation barriers
Trauma Systems Therapy (TST) [115]	Community	USA	Children: In and out-of-home care Age: Mean 11.98 years Adults: Social care/health care professionals	3 years	Not specified	Social care system-wide trauma-informed model of clinical provision and service coordination. Informs decision-making for treatment, training for staff, and system culture
Treatment Foster Care (TFC); Treatment Foster Care (TFC) for Older Youth; Together Facing the Challenge (TFTC) [55, 57, 63, 91, 95, 127, 176]	Interpersonal; Organisational; Community	USA	Children: Foster care Age: < 18 years Adults: Foster carers; Social care/health care professionals	Approx. 12 months	Key staff; Life coach; Psychiatric nurse Care placement; Clinician settings; Community settings	Parenting programme (approx. 2.5 h weekly) including role play and didactic instruction. Range of wraparound services that includes psychiatric support, life skills development, life coaching in education and employment
Triple P for Foster Carers (TPFC) [111]	Interpersonal	Germany	Children: Foster care Adults: Foster carers	5 weeks	Triple P facilitators Community settings	Manualised parenting group (2.5 h weekly), two 20-min telephone consultations and a closure session
Wave by Wave [168]	Intrapersonal; Interpersonal	Portugal	Children: Residential care Age: 10–16 years	6 months	Psychologists; Surf instructors Carcavelos beach, Portugal	Psychoeducation activities and surf classes (3 h weekly sessions)
Youth-Initiated Mentoring (YIM) Relationships [99]	Interpersonal	USA	Children: Foster care Age: 16–25 years	12 months	Mentor from social services, school, church, family or former foster carer Not specified	Youth nominated mentor meets with young person (1 per month) to provide informational, companionship, emotional, appraisal and instrumental support

The majority of interventions (*n* = 26) targeted the interpersonal domain. They primarily focused on the skills, knowledge and confidence of foster and kinship carers through training curricula and professional-delivered support. A small number of interventions promoted children and young people’s positive relationships with biological families, largely with the aim of facilitating reunification [54, 139, 169]. Elsewhere interventions provided opportunities to build relationships with peers [59, 142], trained mentors [99], clinicians [171] and wider social networks [72]. Where details on duration of delivery was specified, most interventions were delivered for 1 to 6 months (*n* = 17). Seven were delivered between 7 and 12 months.

Nine interventions targeted the intrapersonal domain, directly supporting care-experienced children and young people. Approaches included delivery of Cognitive and Affective Bibliotherapy [123], Cognitively-Based Compassion Training (CBCT) [96], Cognitive Behavioural Therapy (CBT) [74, 97] and mindfulness and yoga practices [89, 105]. These were delivered through a range of online and virtual modalities, including online tutorials and computer games [74, 97]. Where specified, interventions were primarily delivered over the course of 1 to 6 months, with only one intervention being delivered for a longer duration than 6 months [74].

A further fifteen interventions operated across the intrapersonal and interpersonal domains, combining both relationship-based components with skill and competency training for children and young people. For example, Fostering Healthy Futures (FHF) provided group-based curricula and mentoring by a trainee social worker [66]. Group-based activities could include creative or leisure tasks, such as drumming [124] or surfing [168]. For some of these interventions, relationships were fostered through animal-facilitated psychotherapy [158], specifically equine therapy [50]. Five interventions were delivered between 1 and 6 months, seven were delivered between 7 and 12 months and one was delivered between 13 and 24 months.

A further eight interventions primarily included intrapersonal and interpersonal targeting activities, but had a range of organisational- and community-based support to reinforce change mechanisms, support linkage to other interventions and optimise delivery. This included Keeping Foster and Kinship Parents Supported and Trained (KEEP) [53, 62, 71, 79, 81, 106, 140, 141, 149, 161], Multidimensional Treatment Foster Care (MTFC) [51–53, 58, 76, 78, 90, 128, 129] and Treatment Foster Care (TFC) [55, 57, 63, 91, 95, 119, 127, 176]. This group of interventions were delivered up to 6 months (*n* = 2), 7–12 months (*n* = 1), 13–24 months (*n* = 2), or delivery duration was not specified (*n* = 3).

There were a limited number of structural-level interventions: one had a focus on organisational culture and ethos [73, 151]; four considered the availability of community mental health and wellbeing provision [80, 115, 131, 147]; and one policy-level approach targeted the re-prioritisation and funding of placement types [150, 153, 157, 159]. Generally, the delivery duration of these interventions were not specified, although one was delivered for 12 months [131] and two for 3 years [73, 115, 151].

### Programme theories

A subset of 13 interventions, with 24 study reports, included a clearly articulated programme theory (Table 2). These mapped onto three dimensions of programme theory: theories of change that explain the causal mechanisms through which an intervention is intended to bring about change; theories of implementation, which prescribe how an intervention will operationalise proposed change mechanisms; and context theories, which consider how system features interact with and are modified by the change mechanisms [177, 178].

**Table 2:** Overview of intervention programme theory (*N* = 13 interventions)

Socio-ecological domain of theory	Intervention	Description of theory	Specified theories
Intrapersonal	Equine-facilitated Psychotherapy [50]	**Physical and mental development:** Horse’s rhythm and riding linked with the mental and physical developmental process	None
Interpersonal		**Therapeutic alliance:** Relationship with horse provides healing experience. Important for building trust with ‘other’ and establishing interpersonal skills	Therapeutic alliance
Intrapersonal	Fostering Healthy Futures [64–66, 69, 70]	**Positive Youth Development:** Need to develop prosocial, behavioural and emotional skills **Resilience theory:** Promote adaptive functioning to increase resiliency	Attachment; Positive Youth Development; Resilience; Social Learning Theory
Interpersonal		**Attachment:** Challenging relationship histories can reduce mentoring responsiveness **Positive Youth Development:** Need to develop prosocial relationships as a template for future relationships **Resilience theory:** Promotes adaptive functioning **Social Learning Theory:** Importance of modelling to develop prosocial relationships	
Intrapersonal	SuppOrting Looked after children In Decreasing Drugs, and alcohol (SOLID) [72]	**Motivational interviewing:** Behavioural and cognitive strategies to support change and remove ambivalence towards substance use	Motivational interviewing
Interpersonal		**Social network support:** Important in supporting young people to deal with problem behaviours and attain goals	None
Interpersonal	Early Intervention Foster Care (EIFC) [56]	**Delayed maturation:** Challenges of children due to delayed maturation. Intervention provides optimal environment to facilitate developmental progress	None
Interpersonal	Foster carer training [61]	**Behavioural management skills:** Parent management training informed by a constructive rather than pathological approach to operant conditioning, which theorises that behaviour can be learned through a system of reward and punishment	Operant conditioning
Interpersonal	Foster parent training [67, 68]	**Attachment Theory:** Care-experienced young people develop mistrust and insecurity because of absent biological caregiver **Social Learning Theory:** Background context theory referenced but not explained	Attachment; Social Learning Theory
Interpersonal	Incredible Years [60]	**Social Learning Theory:** Requirement to alter negative parenting behaviours (e.g. shouting or physical behaviours) modelled to children	Social Learning Theory
Interpersonal	Mentoring intervention for teenage pregnancy [59]	**Attachment:** Need for positive and responsive attachments between the child and caregiver (or mentor and mentee) **Social Learning Theory:** Behaviours learned through the modelling and observation of others	Attachment; Social Learning Theory
Interpersonal	Pathways Home [54]	**Encouragement-based parenting:** Need to develop parenting practices based on encouragement	None
Interpersonal	Keeping Foster Carers Trained and Supported (KEEP) [53, 62, 71]	**Social Learning Theory:** Background context theory referenced but not explained	Social Learning Theory
Organisational/ Community		**Generic System Change:** Cascading dissemination model to support local system capacity	
Interpersonal	Multidimensional Treatment Foster Care (MTFC) [51–53, 58]	**Coercion theory:** Need to prevent negative and coercive interactions between child and their carer and/or peers **Resilience:** Focus on positive, asset-based development **Social Learning Theory/Positive Youth Development:** Background context theory referenced but not explained **Trauma-focused cognitive behavioural therapy:** Carers need to understand and respond to causes of trauma	Coercion Theory; Resilience; Social Learning Theory; Positive Youth Development; Trauma- focused Cognitive Behavioural Therapy
Organisational/ Community		**Generic system change:** Rolling cohort model that commences with learning from small-scale implementation and then applied to wider system	None
Interpersonal	Treatment Foster Care (TFC) [57, 63] / Together Facing the Challenge (TFTC) [55]	**Role enactment:** Encourages carer affiliation with role of parent to support adherence with delivery **Trauma-focused Cognitive Behavioural Therapy:** Carers need to understand and respond to causes of trauma **Therapeutic alliance:** Relationships between the treatment parent and child is therapeutic and the therapeutic relationship provides an environment for positive change **Ecological Context Model:** Intervention nested within larger supra-system of influence that impacts effectiveness: foster carer skills and parenting; contact with biological parents; and relationship with peer group	Trauma-focused Cognitive Behavioural Therapy; Therapeutic Alliance; Ecological Context Model
Organisational/ Community		**Ecological Context Model:** Intervention nested within larger supra-system: lack of educational resources, integration and continuity; delivery agency structure and professionals; funding and access to social services; and young people’s integration into community	
Organisational	Children and Residential Experiences (CARE) [73]	**Generic system change:** Need to transform organisational ethos and culture to create alignment with attachment / relationship-based approach	Attachment; Ainsworth Maternal Sensitivity

Theories of change targeted different socio-ecological domains. Three interventions focused on intrapersonal theories [50, 64–66, 69, 70, 72]. Key theoretical approaches within this domain linked to Positive Youth Development [179] and resilience, emphasising the need for young people’s adaptive functioning and self-development so that they can enter prosocial relationships [64, 66, 69].

The majority of interventions foregrounded interpersonal theories of change (*n* = 12), which mapped onto three sets of causal mechanisms. First was to build a therapeutic environment that could be supportive of positive development and prosocial relationships [50, 56, 63]. Second was to develop parent and carers skills, knowledge and confidence, primarily through parenting curricula [51–58, 60–62, 67, 68]. Theories included Bowlby’s attachment theory [180], Social Learning Theory [181], Positive Youth Development [179] and resilience, which together emphasise the significance of positive attachments that provide opportunities for learning prosocial behaviours observationally through modelling and replication. Some interventions also re-orientated parenting practices according to coercion and operant conditioning, which encourage effective management of negative behaviour through positive reinforcement and non-harsh disciplinary methods [51, 52, 61]. Third, was to develop mentoring relationships [59, 64–66, 69, 101]. These also operated through attachment theory, Social Learning Theory [181] and Positive Youth Development [179].

One intervention included a theory that operated within the organisational domain [73]. The focus was on the transformation of organisational culture within the social care system to ensure its conduciveness with an attachment-based and trauma-informed ethos.

There was more limited inclusion of implementation and context theories. Two interventions operating across the interpersonal, organisation and community domains, focused on optimising delivery in a range of contexts [53, 58, 62, 73]. This included testing a ‘train the trainer’ approach and a structured scale-up model, where the learning from early implementation informed later delivery. We termed these implementation theories as ‘general system change’. One intervention included a context theory, mapping the wider system factors that could inhibit the functioning of an intervention’s parenting curricula. The study report termed this an ‘ecological context model’ [57].

### Intervention outcomes

**Fig. 3:**
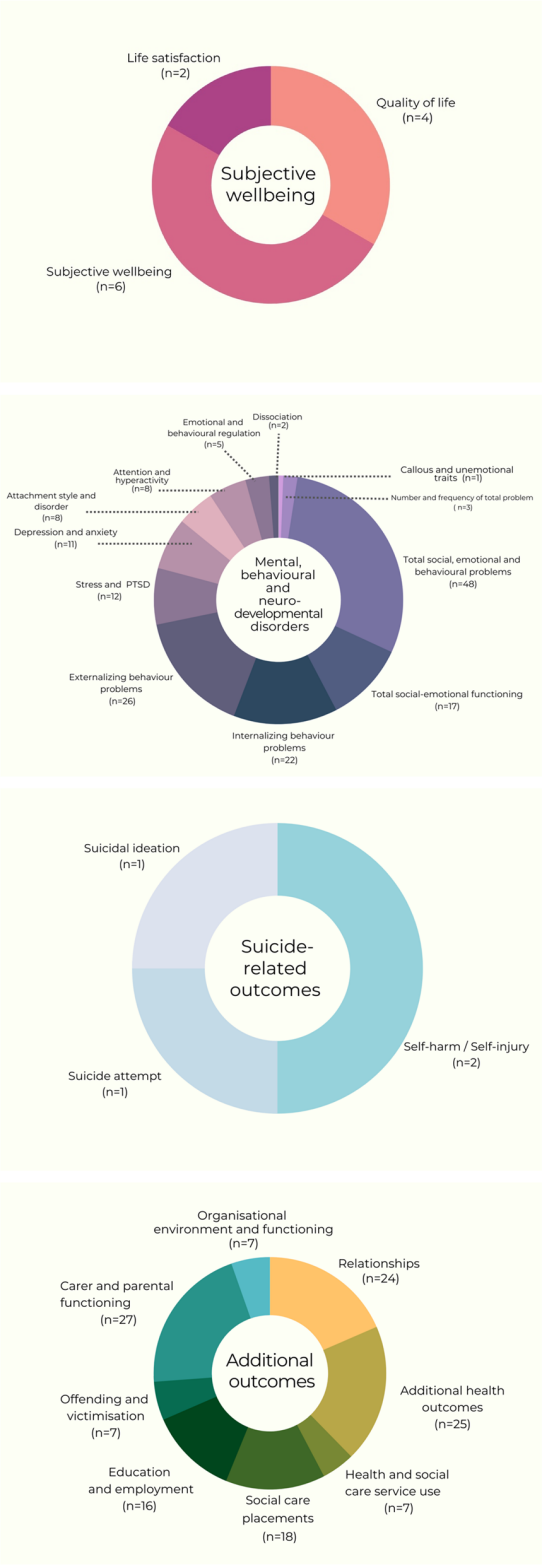
Intervention primary and additional outcome domains

We mapped intervention outcomes according to the a priori outcomes specified by the review (Fig. 3). Outcomes were either theorised (e.g. study reports with theoretical descriptions) or empirically assessed (e.g. study reports with outcome evaluations).

Most interventions targeted mental, behavioural and neurodevelopmental disorders. Within this domain, interventions most frequently assessed outcome measurements of total social, emotional and behavioural problems (*n* = 48); socio-emotional functioning difficulties (*n* = 17); internalising problem behaviours (*n* = 22); and externalising problem behaviours (*n* = 26). There was a paucity of interventions that targeted subjective wellbeing (*n* = 11). Only four interventions targeted suicide-related outcomes, including suicidal ideation [124], self-harm [96, 151] and suicide attempt [59].

We inductively classified additional outcomes measured by evaluations. These were primarily child-level outcomes: relationships; additional health outcomes; health and social care service use; social care placements; education and employment; and offending and victimisation.

We classified fourteen study reports, linked to eight interventions, that considered potential equity harms in relation to intervention outcomes [54, 65, 69–71, 101, 122, 126, 130, 133, 140, 142, 170, 182]. Reported equity harms focused on children and young people’s personal characteristics (age; gender; ethnicity; baseline mental health status) and personal relationships (exposure to maltreatment; placement type; quality of relationship with caregiver; number of caregivers). Parent and carer-related equity harms were linked to personal characteristics (age; ethnicity; baseline mental health status; and drug and alcohol use) and personal relationships (relationship status).

### Evidence types

**Fig. 4:**
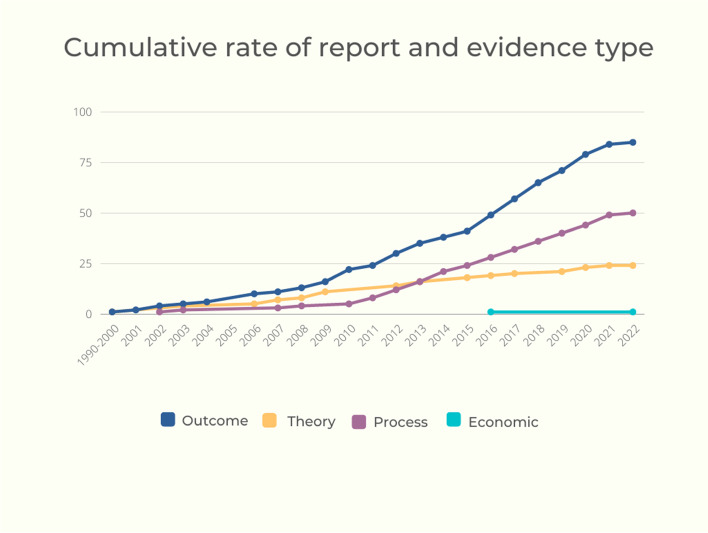
Cumulative rate of report and evidence type

We categorised study reports according to the type of evidence reported (Fig. 4). The evidence type linked to each intervention is further presented in Table 3. Twenty-four study reports described a programme theory [50–73]. Fifty process evaluations provided data on context, implementation and acceptability. Of these, we defined 27 as conceptually and/or empirically ‘thin’, whereby they provided limited description of intervention implementation and acceptability [60, 68, 77–79, 81, 82, 84, 85, 87, 89, 91, 94, 96–98, 101, 102, 106, 111–115, 117, 118, 183]. Meanwhile, 23 were considered conceptually and/or empirically ‘rich’, presenting detailed data and analysis of contextual characteristics that might structure intervention functioning through their influence on implementation and acceptability [72, 74, 80, 83, 86, 93, 95, 99, 100, 103–105, 107–110, 116, 119, 175, 182, 184–186]. This set of rich process evaluations had theoretical generalisability beyond the immediate evaluation context.

**Table 3:** Overview of intervention evidence types

Intervention	Country	Theory	Process	Outcome	
				**RCT**	**Non-randomised**
Acceptance and Commitment Therapy (ACT)	Sweden				Livheim, Tengström, et al*.* (2020) [155]
Animal Associated Psychotherapy (AAP)	Spain				Muela, Balluerka, et al*.* (2017) [158]
Attachment and Behavioural Catch-up (ABC)	USA			Dozier, Peloso, et al*.* (2006) [126]; Sprang (2009) [143]	
Behavioural modification training; psychodynamic treatment; structured community living; adventurous learning	Netherlands				Scholte, Van der Ploeg, et al*.* (2006) [163]
Care placement type	Netherlands; USA				Hayduk (2017) [150]; Leloux-Opmeer, Kuiper, et al*.* (2018) [153]; McCrae, Lee, et al*.* (2010) [157]; Portwood, Boyd, et al*.* (2018) [159]
Child Adult Relationship Enhancement	USA			Messer, Greiner, et al*.* (2018) [135]	Wood, Dougherty, et al*.* (2019) [167]
Child and Adolescent Mental Health Services (CAMHS)	UK		Callaghan, Young, et al*.* (2003) [80]		
Child-Directed Interaction Training (CDIT)	USA			N’Zi, Stevens, et al*.* (2016) [138]	
Children and Residential Experiences (CARE)	USA	Izzo (2020) [73]			Izzo, Smith, et al*.* (2016) [151] Izzo (2020) [73]
Cognitive and Affective Bibliotherapy	Israel			Betzalel, Schechtman (2010) [123]	
Cognitively-Based Compassion Training (CBCT)	USA		Reddy, Negi, et al*.* (2013) [96]	Reddy, Negi, et al*.* (2013) [96]	
Computer game	UK		Aventin, Houston, et al*.* (2014) [74]		
Connect-KP	Australia		Pasalich, Moretti, et al*.* (2021) [117]	Pasalich, Moretti, et al*.* (2021) [117]	
Dojo: Biofeedback videogame	Netherlands		Schuurmans, Nijhof, et al*.* (2018) [97]	Schuurmans, Nijhof, et al*.* (2018) [97]	
Early Intervention Foster Care (EIFC)	USA	Fisher, Ellis, et al*.* (1999) [56]			
Enhanced Foster Care Treatment	USA				Kessler, Pecora, et al*.* (2008) [152]
Equine-Facilitated Psychotherapy (EFP)	Israel	Bachi, Terkel, et al*.* (2012) [50]			Bachi, Terkel, et al*.* (2012) [50]
Evolve Behaviour Support Services (EBSS)	Australia		Ziviani, Darlington, et al*.* (2013) [107]		
Family Finding	USA		Shklarski (2020) [110]	Vandivere, Malm, et al*.* (2017) [146]	Leon, Saucedo, et al*.* (2016) [154]
Family Minds	USA			Adkins, Reisz, et al (2021) [120]	
Foster carer and foster children group-based intervention	USA			Smith, Leve, et al*.* (2011) [142]	
Foster carer training	UK			Minnis, Pelosi, et al*.* (2001) [136]	
Foster carer training	UK	Pithouse, Hill-Tout, et al*.* (2002) [61]			Pithouse, Hill-Tout, et al*.* (2002) [61]
Foster parent training	Belgium	Van-Holen, Vanschoonlandt, et al*.* (2016) [67]; Vanschoonlandt, Vanderfaeillie, et al*.* (2012) [68]	Vanschoonlandt, Vanderfaeillie, et al*.* (2012) [68]	Van-Holen, Vanschoonlandt, et al*.* (2016) [67]	Vanschoonlandt, Vanderfaeillie, et al*.* (2012) [68]
Fostering Changes	UK		Briskman, Castle, et al*.* (2012) [88]	Briskman, Castle, et al*.* (2012) [88]; Moody, Coulman, et al*.* (2020) [137]	
Fostering Connections	Ireland		Lotty, Bantry-White, et al*.* (2020) [92]		Lotty, Dunn, et al*.* (2020) [156]
Fostering Healthy Futures (FHF)	USA	Taussig, Culhane, et al*.* (2007) [66]; Taussig, Culhane, et al*.* (2013) [65]; Taussig, Weiler, et al*.* (2015) [64]; Weiler, Taussig (2019) [70]; Weiler, Lee (2021) [69]	Taussig, Weiler, et al*.* (2019) [101]	Taussig, Culhane (2010) [144]; Taussig, Culhane, et al*.* (2013) [65]; Taussig, Weiler, et al*.* (2019) [101]; Weiler, Taussig (2019) [70]; Weiler, Lee (2021) [69]	
Fostering Individualised Assistance programme (FIAP)	USA			Clark, Prange, et al*.* (1994) [125]	
Glasgow Infant Family Team (GIFT); London Infant Family Team (LIFT); New Orleans Model	UK		Baginsky (2017) [175]; Turner-Halliday, Watson, et al*.* (2016) [104]; Turner-Halliday, Kainth, et al*.* (2017) [103]		
Head, Heart, Hands	UK		McDermid, Trivedi, et al*.* (2021) [93]		
Head Start	USA			Lipscomb, Pratt, et al*.* (2013) [131]	
*HealthRHYTHMS*	USA			Bittman, Dickson, et al*.* (2009) [124]	
Herts and Mind: Mentalization-Based Therapy	UK		Midgley, Besser, et al*.* (2019) [94]	Midgley, Besser, et al*.* (2019) [94]	
Incredible Years	Portugal; UK; USA	Nilsen (2007) [60]	Conn, Szilagyi, et al*.* (2018) [82]; Furlong, McLoughlin, et al*.* (2021) [84]; Hutchings, Bywater (2013) [87]; Nilsen (2007) [60]; Silva, Gaspar, et al*.* (2016) [98]	Conn, Szilagyi, et al*.* (2018) [82]; Linares, Montalto, et al*.* (2006) [130]	Furlong, McLoughlin, et al*.* (2021) [84]; Nilsen (2007) [60]
Individual therapy and rehabilitative strategies	USA				Pozo-Breen (2017) [160]
Intensive Permanence Systems	USA		Hall, Semanchin, et al*.* (2018) [86]		
*k*Contact				Suomi, Lucas, et al*.* (2020) [169]	
Keeping Foster and Kinship Parents Supported and Trained (KEEP)	USA	Chamberlain, Price, et al*.* (2008) [71]; Chamberlain, Roberts, et al*.* (2012) [53]; Price (2009) [62]	Buchanan, Chamberlain, et al*.* (2012) [79]; Walsh (2017) [106]; Chamberlain, Price, et al*.* [81]	Chamberlain, Price, et al*.* (2008) [71]; Price, Roesch, et al*.* (2015) [140]; Price, Roesch, et al*.* (2019) [141]	Greeno, Lee, et al*.* (2016) [149]; Price, Roesch, et al*.* (2012) [161]
Kids in Transition to Schools (KITS)	USA				Bronz (2004) [148]
Kundalini Yoga	UK		Vallejos, Ball, et al*.* (2016) [105]		Vallejos, Ball, et al*.* (2016) [105]
Life Story	USA		Haight, Black, et al*.* (2010) [85]	Haight, Black, et al*.* (2010) [85]	
Mentoring intervention for teenage pregnancy	UK	Mezey, Meyer, et al*.* (2015) [59]	Mezey, Meyer, et al*.* (2015) [59]	Mezey, Meyer, et al*.* (2015) [59]	
Mindfulness	USA		Jee, Couderc, et al*.* (2015) [89]	Jee, Couderc, et al*.* (2015) [89]	
Multidimensional Treatment Foster Care (MTFC); Multidimensional Treatment Foster Care-Adolescents (MTFC-A); Multidimensional Treatment Foster Care-Pre-schoolers (MTFC-P)	Netherlands; UK; USA	Chamberlain (2003) [51]; Chamberlain (2006) [52]; Chamberlain, Roberts, et al*.* (2012) [53]; Leve, Fisher, et al*.* (2009) [58]	Biehal, Dixon, et al*.* (2012) [76]; Brown, Chamberlain, et al*.* (2014) [78]; Kirton, Thomas (2011) [90]	Biehal, Dixon, et al*.* (2012) [76]; Green, Roberts, et al*.* (2014) [128]; Jonkman, Schuengel, et al*.* (2017) [129]	Biehal, Dixon, et al*.* (2012) [76]; Green, Roberts, et al*.* (2014) [128]
NonViolent Resistance (NVR) Training	Belgium			Van-Holen, Vanderfaeillie, et al*.* (2018) [145]	
Opportunities Box	Portugal				Silva, Coelho, et al*.* (2017) [164]
Outpatient mental health services	USA				Bellamy (2013) [147]
Parent–Child Interaction Therapy (PCIT)	USA		Blair, Topitzes, et al*.* (2019) [77]	Mersky, Topitzes, et al*.* (2016) [134]; Mersky, Topitzes, et al*. *(2020) [172]	Timmer, Urquiza, et al*.* (2006) [165]
Parent Management Training (PMT); Parent Management Training Oregon Model (PMTO)	Netherlands; USA		Leathers, Spielfogel, et al*.* (2011) [114]; Maaskant, Van Rooj et al*.* (2016) [118]; Spielfogel, Leathers, et al*.* (2011) [100]	Akin, Lang, Yan, et al*.* (2018) [121]; Akin, Lang, Yan, et al*.* (2019) [122]; Maaskant, Van Rooj et al*.* (2016) [118]; Maaskant, Van Rooj, et al*.* (2017) [132]; Yan & De Luca (2020) [170]	Leathers, Spielfogel, et al*.* (2011) [114]
Pathways Home	USA	DeGarmo, Reid, et al*.* (2013) [54]		DeGarmo, Reid, et al*.* (2013) [54]	
Promoting First Relationships	USA			Oxford, Marcenko, et al*.* (2016) [139]	
Psychosocial rehabilitation	USA				Williams, Sherr (2009) [166]
Sanctuary Model	USA				Rivard, Bloom, et al*.* (2003) [162]
Solution Focused Parenting Group (SFPG)	Canada		Triantafillou (2002) [102]		Triantafillou (2002) [102]
SuppOrting Looked after children In Decreasing Drugs, and alcohol (SOLID)	UK	Alderson, Kaner, et al*.* (2020) [72]	Alderson, Kaner, et al*.* (2020) [72]; Alderson, Kaner, et al*.* (2020) [108]; Alderson, McGovern, et al*.* (2021) [109]	Alderson, Kaner, et al*.* (2020) [72]; Alderson, Kaner, et al*.* (2020) [108]	
TAKE CHARGE	USA		Geenen, Powers, et al*.* (2013) [113]	Geenen, Powers, et al*.* (2013) [113]	
Teach Your Children Well	Canada			Marquis (2014) [133]	
Therapeutic Mentoring	USA				Johnson, Price et al*.* (2010) [171]
Trauma-Focused Cognitive Behavioural Therapy (TF-CBT) and evidence-based engagement strategies	USA		Dorsey, Conover, et al*.* (2014) [83]; Dorsey, Pullmann, et al*.* (2014) [112]	Dorsey, Pullmann, et al*.* (2014) [112]	
Trauma Systems Therapy (TST)	USA		Murphy, Moore, et al*.* (2017) [115]		Murphy, Moore, et al*.* (2017) [115]
Treatment Foster Care (TFC); Treatment Foster Care (TFC) for Older Youth; Together Facing the Challenge (TFTC)	USA	Farmer, Lippold (2016) [55]; James, Meezan (2002) [57]; Southerland, Mustillo et al. (2009) [63]	Lee, Phillips, et al*.* (2021) [91]; Tullberg, Vaughon, et al*.* (2019) [176]; McMillen, Narendorf, et al*.* (2015) [119]; Murray, Culver, et al*.* (2014) [95]	Farmer, Burns, et al*.* (2010) [127]	
Triple P for Foster Carers (TPFC)	Germany		Job, Ehrenberg, et al*.* (2020) [111]	Job, Ehrenberg, et al*.* (2020) [111]	
Wave by Wave	Portugal			Pereira, Ferreira, et al*.* (2020) [168]	
Youth-Initiated Mentoring (YIM) Relationships	USA		Spencer, Drew, et al*.* (2018) [99]		

There were 86 outcome evaluations. Of these, 52 were randomised controlled trials (RCTs) and 34 were non-randomised evaluations. The majority of RCTs (*n* = 43) evaluated interventions (*n* = 31) that primarily targeted the intrapersonal or interpersonal domains [54, 59, 65, 67, 69, 70, 82, 85, 88, 89, 94, 96, 97, 101, 111–113, 117, 118, 120–124, 126, 130, 132–139, 142–146, 168–170, 172]. Five interventions that operated across the organisational, community and policy domains were evaluated via an RCT (*n* = 9 study reports) [71, 125, 127–129, 131, 140, 141, 182]. Of interventions evaluated through a non-randomised study, 21 interventions, with 22 study reports, targeted the intrapersonal and interpersonal domains [50, 60, 61, 68, 84, 102, 105, 114, 148, 152, 154–156, 158, 160, 162–167, 171]. Six interventions, with 12 evaluations targeted the organisational, community and policy domains [73, 115, 128, 147, 149–151, 153, 157, 159, 161, 182]. There were 14 study reports that provided moderator analysis or interaction effects that were relevant to assessing equity harms [54, 65, 69–71, 101, 122, 126, 130, 133, 140, 142, 170, 182].

There was one partial economic evaluation, which estimated the relative costs and consequences of a new intervention compared to the estimated costs of usual care [173].

## Discussion

The CHIMES systematic review aimed to synthesise international evidence on interventions targeting the mental health and wellbeing of care-experienced children and young people. The first phase of the review, an evidence map of the available literature, is reported presently.

Mapping interventions by the socio-ecological domain targeted, the main cluster was intrapersonal and interpersonal approaches, often targeting children and young people’s skills and knowledge, or carers’ parenting practices. Some of these also combined organisational and community facing activities to optimise functioning and implementation. As identified in a range of systematic and practitioner reviews of parenting interventions for care-experienced children and young people [187–190], a couple of interventions were dominant in the map. These were the USA originated Multidimensional Treatment Foster Care (MTFC) [51–53, 58, 76, 78, 90, 128, 129] and its derivative Keeping Foster and Kinship Parents Supported and Trained (KEEP) [53, 71, 62, 79, 106, 140, 141, 149, 161], which provide intensive parenting training for foster and kinship carers, embedded in a wider system of support services. Overall, these interventions were under-described and under-theorised, but where specified they often draw on theories related to social modelling and prosocial developmental contexts [179, 181].

In contrast, there was a clear gap in structural-level interventions targeting organisational, community and policy drivers. This is significant given that risk factors for poor mental health in this population include a constellation of family and child welfare system-level factors, which are embedded in a wider context of community-level challenges, such as economic opportunity and socio-economic deprivation [191]. Equally, structural interventions were identified as a priority area for stakeholders who informed the scope and focus of the CHIMES review.

The map identified a wealth of interventions targeting mental health, behavioural and neurodevelopmental disorders, specifically total social, emotional and behavioural problems. Conversely, there was a dearth of interventions targeting subjective wellbeing and suicide-related outcomes, despite care-experienced young people reporting relative adversity in these areas compared to the general population [7, 8]. This reflects wider findings in the research evidence, with a recent review of suicide prevention interventions for children involved in child protection services also identifying a paucity of evidence-based approaches [192]. New interventions might be developed to target these outcomes, or existing approaches adapted if theoretically appropriate. To this end, there is a need to further develop the operationalisation of these constructs and understand the causes that should be targeted to leverage the most change [193]. The extant evidence base, while limited, suggests potential drivers of wellbeing that might be targeted. Primarily operating within the interpersonal domain, these include positive relationships with teachers and family [7, 194], and having available supports, notably material support [194]. Causal mechanisms for suicide-related outcomes are less evident, with current research tending to focus on identifying socio-demographic risk profiles within this population (e.g. age, ethnicity and maltreatment exposure) [6].

The evidence map has implications for future research. Presently the weight of available evidence is focused on outcome evaluation, although only a limited number consider the potential for equity harms [195]. Methodological guidance related to the development, adaptation and evaluation of interventions recommends the integration of outcome data with a clear understanding of the underpinning theory, explication of context, implementation and acceptability through process evaluation, and economic evaluation [39, 40, 196].

As indicated, there remains a lack of description of interventions’ programme theory, with less than a fifth of included interventions reporting a theoretical basis. This is imperative in knowing how interventions interact with system conditions in the generation of outcomes. Given that the evidence base is predominantly located in the USA to date, this means that there is currently a lack of knowledge about the implementation of different approaches in diverse contexts, cultures and countries. Understanding how programme theories function in the USA evaluation context can offer insight into the potential replicability of effects elsewhere. It can then support efforts to adapt interventions to different settings or population subgroups, or to identify where transportation may not be suitable and new approaches need to be developed [39].

Equally, while there continues to be expansion in the conduct of process evaluations, these tend to be conceptually and empirically thin, providing rudimentary summaries of reach and delivery. This is reflected in systematic reviews that currently synthesise process evaluation data, which largely detail barriers and facilitators to implementation [26]. Understanding of wider contextual characteristics, through conceptually and empirically rich process evaluation, is important from a complex systems perspective, which emphasises that intervention’s functioning is dependent on its interaction with both proximal and distal system characteristics [30–34].

There is also a paucity of economic evaluations, which reflects a wider issue identified in children’s social care research [197]. Failing to attend to the cost-effectiveness of interventions is a particular concern given extant issues around escalating costs across social care systems [14].

Beyond implications for evaluation research, there are also some initial suggestions for enhancing systematic reviews in the area of care-experienced populations. It is important that interventions are more comprehensively described in evaluations, preferably with the use of reporting guidance such as the TIDIeR Framework for intervention descriptions [47]. Systematic description of the complex system in which interventions are delivered, using frameworks such as the Context and Implementation of Complex Interventions (CICI) framework [198], will be particularly helpful in supporting future syntheses. In regard to the review process, future reviews of intervention evaluations might aim to map and synthesise all relevant types of evidence [47], particularly in relation to theory, equity and economic outcomes. This will help to identify where gaps continue and where good practice is emerging. Finally, reviews might take advantage of methodological progress in integrating complex systems perspectives into systematic reviews, to help understand the interaction of interventions with system features more fully [34, 199].

## Review limitations

There are five central limitations associated with the evidence map. First, the literature around care-experienced populations can be challenging to identify, largely due to extensive international variations in terminology. As such, while the review searches were designed and tested to be sensitive, some study reports may have been missed. Second, the review was limited to studies conducted in higher-income countries, as classified by the Organisation for Economic Co-operation and Development (OECD). As a result, the review has limited generalisability to middle- and lower-income countries, and potentially higher-income countries that do not fall within this classification. Third, there was limited reporting of interventions and associated evaluations, which provided challenges in the cataloguing and mapping of study reports. There were further issues due to the under-specification of interventions’ programme theory. Fourth, study reports were aggregated to chart overarching evidence gaps and clusters. As a result, some of the diversity between interventions and countries is not fully described. Fifth, at the stage of evidence mapping, we did not quality appraise study reports. As such reporting of evidence clusters only reflects the quantity of interventions and evaluations.

## Conclusion

The present evidence map describes intervention and evidence clusters and gaps in relation to mental health and wellbeing interventions for care-experienced children and young people. With the predominance of intrapersonal and interpersonal interventions from the USA, future development and adaptation might focus on structural-level theories and components, paying attention to how they function in different contexts. They might also focus on subjective wellbeing and suicide-related outcomes. Intervention research needs to integrate theory, outcome, process and economic evaluation to strengthen the evidence base.

## Data Availability

Data extraction, analysis and synthesis are available from the corresponding author on reasonable request.
